# Modulation of Osteogenic Gene Expression by Human Osteoblasts Cultured in the Presence of Bisphenols BPF, BPS, or BPAF

**DOI:** 10.3390/ijms24054256

**Published:** 2023-02-21

**Authors:** Enrique García-Recio, Víctor J. Costela-Ruiz, Rebeca Illescas-Montes, Lucía Melguizo-Rodríguez, Olga García-Martínez, Concepción Ruiz, Elvira De Luna-Bertos

**Affiliations:** 1 Biomedical Group (BIO277), Department of Nursing, Faculty of Health Sciences, University of Granada, Avda. Ilustración 60, 18016 Granada, Spain; 2 Institute of Biosanitary Research, ibs.GRANADA, Avda. de Madrid, 15 Pabellón de Consultas Externas, 2a Planta, 18012 Granada, Spain; 3 Institute of Neuroscience, University of Granada, 18016 Granada, Spain

**Keywords:** bisphenol S, bisphenol F, bisphenol AF, osteoblast, gene expression

## Abstract

Bone effects attributed to bisphenols (BPs) include the inhibition of growth and differentiation. This study analyzes the effect of BPA analogs (BPS, BPF, and BPAF) on the gene expression of the osteogenic markers RUNX2, osterix (OSX), bone morphogenetic protein-2 (BMP-2), BMP-7, alkaline phosphatase (ALP), collagen-1 (COL-1), and osteocalcin (OSC). Human osteoblasts were obtained by primary culture from bone chips harvested during routine dental work in healthy volunteers and were treated with BPF, BPS, or BPAF for 24 h at doses of 10^−5^, 10^−6^, and 10^−7^ M. Untreated cells were used as controls. Real-time PCR was used to determine the expression of the osteogenic marker genes RUNX2, OSX, BMP-2, BMP-7, ALP, COL-1, and OSC. The expression of all studied markers was inhibited in the presence of each analog; some markers (COL-1; OSC, BMP2) were inhibited at all three doses and others only at the highest doses (10^−5^ and 10^−6^ M). Results obtained for the gene expression of osteogenic markers reveal an adverse effect of BPA analogs (BPF, BPS, and BPAF) on the physiology of human osteoblasts. The impact on ALP, COL-1, and OSC synthesis and therefore on bone matrix formation and mineralization is similar to that observed after exposure to BPA. Further research is warranted to determine the possible contribution of BP exposure to the development of bone diseases such as osteoporosis.

## 1. Introduction

The term endocrine disruptor refers to a set of chemicals with a specific effect on the endocrine system that interferes with receptor-mediated hormone activity. Endocrine-disrupting chemicals (EDCs) are substances capable of altering cellular metabolism and causing long-term toxic effects. This group includes molecules of natural origin and synthetic compounds [1,2]. Bisphenols are compounds that are widely found in nature as they are used in the manufacture of plastics and resins. Since they are EDCs, they can imitate or block hormone receptors, altering the concentration and metabolism of hormones and exerting adverse effects at different levels of the organism [3]. Due to widespread exposure and concern that BPA was a toxicant affecting, among other areas, reproductive health, health authorities urged manufacturers to abandon the use of BPA and introduce similar safer chemicals. BPA analogs (BPF, BPS, and BPAF) were designed to replace BPA in the manufacture of certain materials and utensils, especially those in contact with food or used in the home, to avoid the known toxicity of this compound. However, various studies have found that the toxicity of these analogs is similar to that previously observed for BPA [4,5,6,7,8].

BPA has a harmful effect on bone tissue since it can interact via estrogenic receptors with the two main bone tissue populations, osteoblasts and osteoclasts, possibly compromising bone health [1]. BPA inhibits the growth of murine (MC3T3-E1 cell line) [9] and human fetal (hFOB 1.19 cell line) osteoblasts [10] and human osteoblasts obtained by primary culture from bone implants [11]. This growth inhibition results from apoptosis induction, which compromises cell viability in a dose-dependent manner. BPA can also affect the function of this cell population by inhibiting alkaline phosphatase (ALP) synthesis and therefore the mineralization process [10,11,12]. BPA treatment has been found to alter the gene expression of RUNX2, osterix (OSX), β-catenin, collagen-1 (COL-1), osteocalcin (OSC), and bone morphogenetic proteins (BMPs) 2 and 7 (BMP-2 and BPM-7) [9,10,11,13], and the expression of these osteogenic markers is associated with the differentiation and maturation and, therefore, functional capacity of osteoblasts [14].

Given that the presence of BPA has been found to modulate the growth and function of human osteoblasts, the objective of this study was to determine the effect of osteoblast culture with BPF, BPS, or BPAF on the gene expression of RUNX2, ALP, OSX, COL-1, OSC, BMP-2, and BMP-7.

## 2. Results

Quantitative RT-PCR (q-RT-PCR) results for the gene expression of osteoblasts after 24 h of culture with BPS, BPF, and BPAF at doses 10^−5^, 10^−6^, and 10^−7^ M were compared with results for untreated control cells.

### 2.1. RUNX2

**Figure 1:**
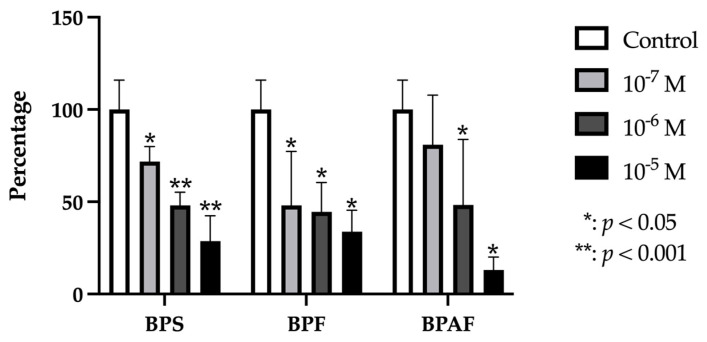
Expression of RUNX2 in primary human osteoblasts treated for 24 h with bisphenol S, F, and AF (10^−7^, 10^−6^, or 10^−5^ M). The assay was performed in triplicate with each of the three primary human osteoblast cell lines. Data are expressed as percentage expression with respect to control ± standard deviation. Significant differences * *p* < 0.05; ** *p* < 0.001.

RUNX2 gene expression was significantly and dose-dependently reduced by treatment with each BP (BPF, BPS, or BPAF) at each dose except for BPAF, which produced no significant change in expression at the lowest dose (10^−7^ M) (Figure 1).

### 2.2. BMP-2 and BMP-7

**Figure 2:**
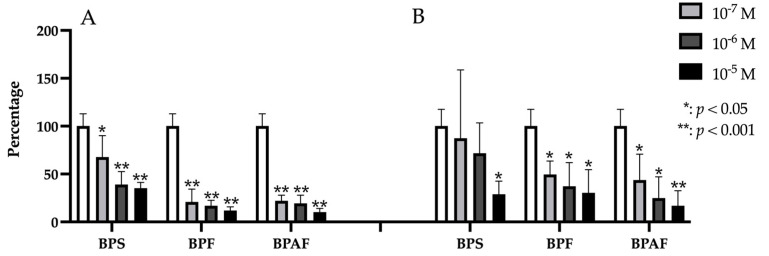
Expression of BMP2 (**A**) and BMP7 (**B**) in primary human osteoblasts treated for 24 h with bisphenol S, F, and AF (10^−7^, 10^−6^, or 10^−5^ M). The assay was performed in triplicate with each of the three primary human osteoblast cell lines. Data are expressed as percentage expression with respect to control ± standard deviation. Significant differences * *p* < 0.05; ** *p* < 0.001.

Gene expression of both BMP2 and BMP7 was significantly and dose-dependently decreased by treatment with each BP at each dose except for the lowest dose of BPS (10^−7^ M), which produced no significant change in expression (Figure 2).

### 2.3. OSX

**Figure 3:**
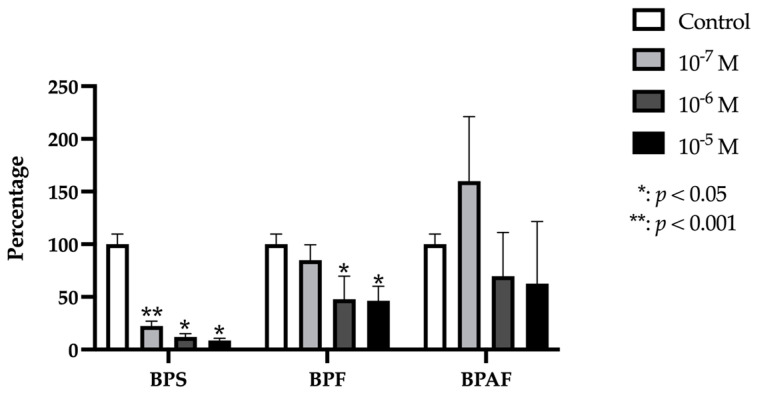
Expression of OSX in primary human osteoblasts treated for 24 h with bisphenol S, F, and AF (10^−7^, 10^−6^, or 10^−5^ M). The assay was performed in triplicate with each of the three primary human osteoblast cell lines. Data are expressed as percentage expression with respect to control ± standard deviation. Significant differences * *p* < 0.05; ** *p* < 0.001.

OSX gene expression was significantly and dose-dependently reduced by treatment with each dose of each BP except for BPAF, which had no significant effect at any dose (Figure 3).

### 2.4. ALP

**Figure 4:**
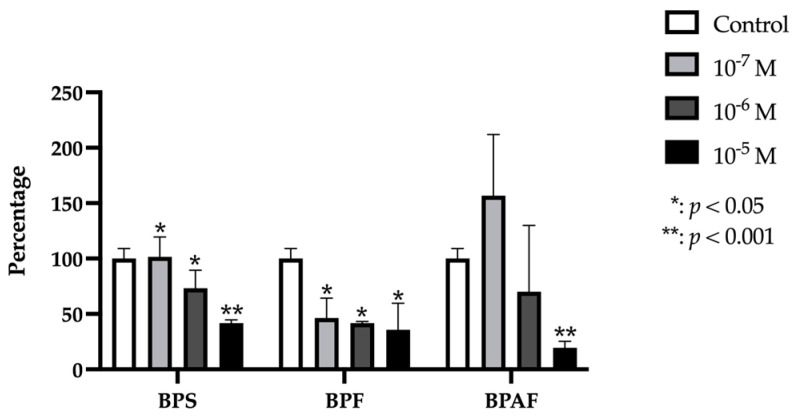
Expression of ALP in primary human osteoblasts treated for 24 h with bisphenol S, F, and AF (10^−7^, 10^−6^, or 10^−5^ M). The assay was performed in triplicate with each of the three primary human osteoblast cell lines. Data are expressed as percentage expression with respect to control ± standard deviation. Significant differences * *p* < 0.05; ** *p* < 0.001.

ALP gene expression was significantly and dose-dependently reduced by treatment with each dose of BPF and BPS, but was only significantly decreased by treatment with the highest dose of BPAF (Figure 4).

### 2.5. OSC

**Figure 5:**
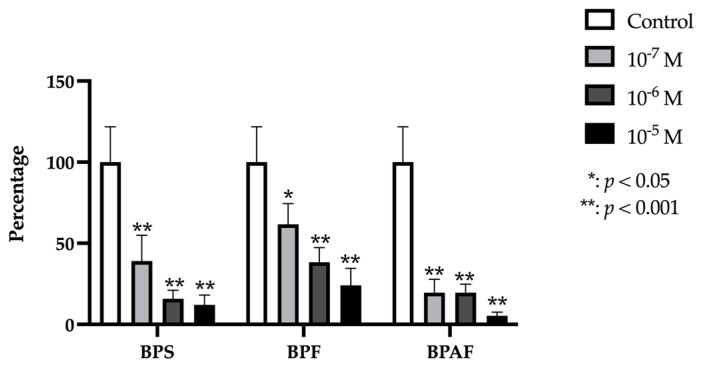
Expression of OSC in primary human osteoblasts treated for 24 h with bisphenol S, F, and AF (10^−7^, 10^−6^, or 10^−5^ M). The assay was performed in triplicate with each of the three primary human osteoblast cell lines. Data are expressed as percentage expression with respect to control ± standard deviation. Significant differences * *p* < 0.05; ** *p* < 0.001.

OSC gene expression was significantly and dose-dependently decreased by each BP at each dose (Figure 5).

### 2.6. COL-1

**Figure 6:**
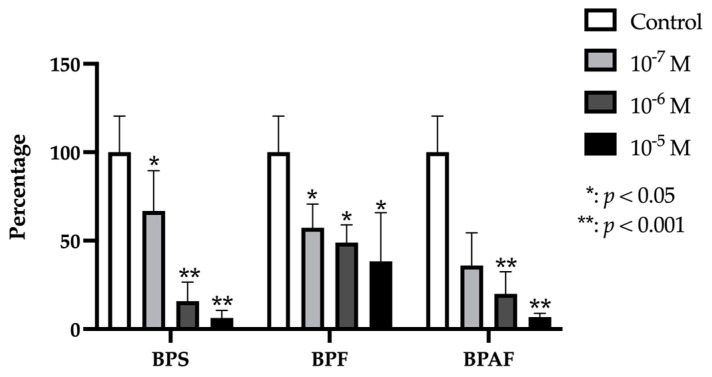
Expression of Col-1 in primary human osteoblasts treated for 24 h with bisphenol S, F, and AF (10^−7^, 10^−6^, or 10^−5^ M). The assay was performed in triplicate with each of the three primary human osteoblast cell lines. Data are expressed as percentage expression with respect to control ± standard deviation. Significant differences * *p* < 0.05; ** *p* < 0.001.

Col-1 gene expression was significantly and dose-dependently reduced by treatment with each dose of BPF and BPS, but BPAF produced a significant change in expression at the higher doses only (10^−5^, and 10^−6^ M) (Figure 6).

## 3. Discussion

In this study, human osteoblasts cultured for 24 h in the presence of BPA analogs (BPF, BPS, or BPAF) underwent significant changes in the gene expression of RUNX2, OXS, OSC, ALP, COL-1, BMP-2, and BMP-7, which are osteogenic markers with key roles in osteoblast maturation and function [15,16,17]. Culture with these BPA analogs was found to inhibit the expression of these markers in a dose-dependent manner.

Mesenchymal cell-based osteogenic differentiation is regulated by various transcriptional factors (e.g., RUNX-2/Cbfa, BMP, and OSX) that are essential for regulating the genes involved in the production of bone extracellular matrix proteins (e.g., ALP, COL-I, bone sialoprotein [BSP], OSC, and osteopontin [OPN]) and for inducing bone mineralization [18,19,20]. RUNX-2/Cbfa, BMP, and OSX genes participate in the formation and differentiation of osteoblasts by activating signals that favor the production of molecules closely related to bone metabolism [17,21]. Each stage of osteoblast functional differentiation (proliferation, bone matrix synthesis, and mineralization) is associated with certain cell markers [19]. Specifically, OSX and RUNX2 are expressed in immature osteoblasts and maintain their expression throughout the osteogenic lineage [20,22], while BMP-2 and BMP-7 are both related to osteoblast formation and differentiation [23,24,25,26], and the major inhibition of their expression can halt the differentiation process [27]. In the present study, the presence of BPF, BPS, or BPAF was found to inhibit the gene expression of BMP-2, BMP-7, RUNX2, and OSX.

The comparison with published findings on the expression of osteogenic markers by human osteoblasts cultured in the presence of BPA [11] shows that culture with BPS has similar effects but that culture with BPF inhibits a larger number of osteogenic markers at all three doses (10^−5^, 10^−6^, and 10^−7^ M). In the case of BPAF, higher doses are required to alter the expression of these osteogenic markers, while the expression of OSX is not changed at any of the three doses assayed. Given the functional involvement of these markers in the osteoblast lineage, not only BPA but also its analogs BPF, BPS, and BPAF may compromise osteoblast differentiation and maturation. This could have undesirable repercussions on the complex process of bone development, as these markers play a crucial role in the molecular mechanism of osteogenesis.

The observation of a decrease in the expression of RUNX2, OXS, and BPMs in human osteoblasts cultured in the presence of BPF, BPS, or BPAF helps to explain the reduced expression of the osteogenic markers ALP, OSC, and COL-1, which play a major role in the formation and mineralization of the bone tissue extracellular matrix. ALP is a metalloenzyme that hydrolyzes monophosphate esters at alkaline pH (8–10), thereby releasing the inorganic phosphorus required for bone mineralization [28]. ALP is expressed at an early stage of osteoblast differentiation, being present on the cell surface and in bone matrix vesicles. Its expression is subsequently reduced during osteoblast maturation, when other genes (e.g., OSC) are upregulated [29]. OSC is a late gene expression that encodes the homonymous peptide hormone synthesized by osteoblasts, which is the main non-collagen bone tissue protein. It contributes to bone conformation and mineralization by favoring the ordered deposit of minerals through regulation of the amount and size of hydroxyapatite crystals. Hence, the main function of OSC is the regulation of matrix synthesis [30,31]. Finally, COL-1, which is the most abundant protein in the bone matrix, is synthetized by mature osteoblasts and plays a structural role. Its expression is observed in nodules mineralized in vitro and mainly in the mature matrix of bone in vivo. COL-1 is considered an osteoblast-specific marker, despite being expressed by cells that are not of osteogenic lineage [32].

Bone remodeling is an active process crucial to adult bone homeostasis that involves a balanced coordination of bone formation and resorption to maintain bone mass and systemic mineral homeostasis. Therefore, the balance of this process guarantees bone health, and any factor that alters it, whether endogenous or exogenous, compromises bone health. Under normal conditions, 5–10% of the total bone is renewed every year. In bone remodeling, the osteoclasts resorb a certain amount of bone and the osteoblasts form the osteoid matrix and mineralize it to fill the cavity previously created. It is therefore a complex process in which cellular and molecular components are closely associated. The cells closely involved (osteoclasts, preosteoblasts, and osteoblasts) are governed by a series of molecular signals that will allow the normal functioning of the bone and the maintenance of bone mass. When this process loses its balance, bone pathology appears, either by excess (osteopetrosis) or by defect (osteoporosis) [33,34].

The changes in the gene expression of ALP, COL-1, and OSC in culture with the three BPs under study suggest that these compounds have a negative impact on the bone extracellular matrix, similar to the reported effects of BPA at osteoblast level. This effect on the expression of these markers is closely related to the inhibition of mineralization and ALP activity observed in human osteoblasts cultured in osteogenic medium in the presence of BPA [11]. Human exposure to bisphenols is widespread in the general population and specifically in workers handling these substances [35]. The ubiquity of BPs indicates that this exposure occurs through food intake, drinking water, by contact of skin with thermal paper, or by dust inhalation [36,37,38,39]. It has been observed that 75% of 267 foods tested had bisphenols in concentrations ranging from 0.10 ng/g fresh weight to 1130 ng/g fresh weight [38]. Moreover, the presence of these bisphenols has been determined in human urine, serum and breast milk samples [40,41,42,43]. The reported presence of BPA analogs in human biological samples, coupled with the close structural similarity to BPA, suggests a possible adverse effect on the organism, as described for BPA, which has already been documented in other tissues. In this regard, our findings suggest that lifetime exposure to BPs could represent a possible risk factor for the development of osteoporosis, a disease of increasing prevalence [44]. Given the importance of the potential risks of exposure to these BPs, it is necessary to study in depth the impact of these molecules on bone tissue, both in vitro and in vivo.

## 4. Materials and Methods

### 4.1. Chemicals

BPF, BPS, and BPAF were obtained from Sigma-Aldrich (St. Louis, MO, USA) and dissolved in dimethyl sulfoxide (DMSO) to a final DMSO concentration of ≤0.05%.

### 4.2. Isolated and Primary Culture of Human Osteoblasts

Primary human osteoblasts were obtained from trabecular bone chips harvested during routine mandibular osteotomy or third molar extraction in healthy individuals at the Clinic of the School of Odontology at our university. Three patients were recruited for this trial from which three primary human osteoblasts cell lines were established. All individuals signed their informed consent to participation in the study, which was approved by the university research ethics committee (Reg. No. 523/CEIH/2018). Osteoblasts were isolated, characterized, and cultured as described by García-Martínez et al. (2011) and Melguizo-Rodríguez et al. (2018) [45,46]. Bone fragments were washed thoroughly in phosphate-buffered saline solution (PBS, pH 7.4) and were seeded onto culture dishes (Falcon Labware, Oxford, UK). They were covered with complete culture medium [Dulbecco’s-modified Eagle medium (DMEM; Invitrogen Gibco Cell Culture Products, Carlsbad, CA, USA) supplemented with 100 IU/mL penicillin (Lab ERN SA, Barcelona, Spain), 50 μg/mL gentamicin (Laboratorios Normon SA, Madrid, Spain), 2.5 μg/mL amphotericin B (Sigma, St. Louis, MO, USA), 1% glutamine (Sigma, St. Louis, MO, USA), 2% HEPES (Sigma, St. Louis, MO, USA), and 20% fetal bovine serum (FBS; Gibco, Paisley, UK)].

Cells were kept in a humidified atmosphere of 95% air and 5% CO_2_ and at 37 °C. After reaching confluence (2–3 weeks), cells were detached from the culture flask with a solution of 0.05% trypsin (Sigma, St. Louis, MO, USA) and 0.02% ethylene-diamine tetraacetic acid (EDTA; Sigma, St. Louis, MO, USA) and were washed and suspended in complete culture medium with 20% FBS.

### 4.3. Treatments

Osteoblasts obtained were treated for 24 h with BPS, BPF, or BPAF (Sigma-Aldrich) at doses of 10^−5^, 10^−6^, or 10^−7^ M; untreated cells were used as controls.

### 4.4. Effect of BPF, BPS, and BPAF on the Gene Expression of Human Osteoblasts

Real-time polymerase chain reaction (RT-PCR) was used to determine the effect of BPs on the gene expression of cultured human osteoblasts. After 24 h of culture with the BP at the corresponding dose, an 0.05% trypsin–EDTA solution (Sigma) was used to detach the cells. The Qiagen RNeasy kit (Qiagen Inc., Hilden, Germany) was used for mRNA extraction in accordance with kit instructions. The amount of mRNA extracted was measured by UV spectrophotometry at 260 nm (Eppendorf AG, Hamburg, Germany), and contamination with proteins was determined according to the 260/280 ratio. Next, 1 μg mRNA from osteoblasts cultured with each BP at each dose was brought to a total volume of 40 μL and reverse-transcribed to cDNA and amplified by PCR using the iScript^TM^ cDNA Synthesis Kit (Bio-Rad laboratories, Hercules, CA, USA) in accordance with the manufacturer’s instructions [47].

RT-PCR primers were designed using the NCBI nucleotide library and Primer3 design to detect mRNA of Runx-2, OSX, ALP, OSC, Col-I, BMP-2, or BMP-7 (Table 1). Ubiquitin C (UBC), peptidylprolyl isomerase A (PPIA), and ribosomal protein S13 (RPS13) were used as stable housekeeping genes to normalize the results [48].

Quantitative RT-PCR (q-RT-PCR) was performed with the SsoFast^TM^ EvaGreen^®^ Supermix Kit (Bio-Rad laboratories), placing cDNA samples in 96-well microplates and using IQ5-Cycler (Bio-Rad laboratories) to amplify the genetic information. More than 40 cycles were performed with annealing temperatures ranging from 60 to 65 °C and an elongation temperature of 72 °C. PCR reactions were performed in a total volume of 20 μL, including 5 μL from cDNA samples and 2 μL from the primer. Standard curves were constructed for each gene by plotting Ct values against log cDNA dilution. Nonspecific PCR products and primer dimers were then excluded by creating a melting profile and performing agarose gel electrophoresis. mRNA concentrations for each gene were expressed as ng of mRNA per average ng of housekeeping mRNA [49]. This assay was performed in triplicate.

### 4.5. Statistical Analysis

mRNA levels were expressed as means ± SD. The normality of variable distributions was checked with the Kolmogorov–Smirnov test. Data were analyzed using ANOVA with Bonferroni corrections for multiple comparisons. Three cell lines of primary culture human osteoblasts were employed for all experiments, performing at least three experiments in all assays. SPSS 22.0 (IBM, Chicago, IL, USA) was used for data analyses, with *p* < 0.05 considered to be significant in all tests.

## 5. Conclusions

This in vitro study demonstrates that BPA analogs (BPF, BPS, and BPAF) exert adverse effects on the expression of osteogenic markers involved in bone development and may inhibit the formation and mineralization of the bone matrix, with a potentially negative impact on the biomechanical properties of bone. The effects observed for these BPA analogs (BPF, BPS, and BPAF) were not substantively different from those previously reported for BPA itself, suggesting that the utilization of these analogs should be subject to comparable supervision and control measures. Further research is warranted to determine the possible contribution of BP exposure to the development of bone diseases such as osteoporosis.

## Figures and Tables

**Table 1:** Primer sequences for the amplification of osteoblasts’ cDNA by RT-PCR.

Gene	Sense Primer	Antisense Primer
RUNX-2	5′-TGGTTAATCTCCGCAGGTCAC-3′	5′-ACTGTGCTGAAGAGGCTGTTTG-3′
BMP-2	5′-TCGAAATTCCCCGTGACCAG-3′	5′-CCACTTCCACCACGAATCCA-3′
BMP-7	5′-CTGGTCTTTGTCTGCAGTGG-3′	5′-GTACCCCTCAACAAGGCTTC-3′
OSX	5′-TGCCTAGAAGCCCTGAGAAA-3′	5′-TTTAACTTGGGGCCTTGAGA-3′
ALP	5′-CCAACGTGGCTAAGAATGTCATC-3′	5′-TGGGCATTGGTGTTGTACGTC-3′
OSC	5′-CCATGAGAGCCCTCACACTCC-3′	5′-GGTCAGCCAACTCGTCACAGTC-3′
COL-I	5′-AGAACTGGTACATCAGCAAG-3′	5′-GAGTTTACAGGAAGCAGACA-3′

## Data Availability

The data presented in this study are available on request from the corresponding author.
